# A comparison of univariate, bivariate, and trivariate whole-genome linkage screens of genetically correlated electrophysiological endophenotypes

**DOI:** 10.1186/1471-2156-6-S1-S117

**Published:** 2005-12-30

**Authors:** Diane M Warren, Thomas D Dyer, Charles P Peterson, Michael C Mahaney, John Blangero, Laura Almasy

**Affiliations:** 1 Department of Genetics, Southwest Foundation for Biomedical Research, P.O. Box 760549, San Antonio, Texas 78245-0549 USA

## Abstract

We used a maximum-likelihood based multipoint linkage approach implemented in SOLAR to examine simultaneously linkage for three electrophysiological endophenotypes from the Collaborative Study of the Genetics of Alcoholism: TTTH1, TTTH2, and TTTH3. These endophenotypes have been identified as markers of alcohol dependence susceptibility. Data were from 905 individuals in 143 families. Measured covariates considered included sex, age at electrophysiology data collection, habitual smoking status, and the maximum number of drinks consumed in a 24-hour period. Comparisons were made among genome-wide univariate, bivariate, and trivariate linkage analyses using genotypes based on microsatellite markers supplied by the Center for Inherited Disease Research, and genotypes based on single-nucleotide polymorphism markers provided by Illumina. All LODs were corrected to a standard equivalent to 1 degree of freedom. Using the trivariate approach and the microsatellite-based genotypes, we estimated a maximum multipoint linkage signal of LOD = 2.66 on chromosome 7q at 157 cM. Analyses using the Illumina SNP genotypes produced similar results, yielding a maximum multipoint LOD of 2.95 on 7q at 174 cM. These regions of interest correspond to those identified in the univariate and bivariate linkage screens. Our results suggest that trivariate multipoint linkage analyses have utility in the further characterization of chromosomal regions potentially containing genes influencing the phenotypes being examined. Based on a comparison of the number of LOD scores achieving statistical significance, our results suggest that the microsatellite- and Illumina SNP-based genotypes have similar utility for detecting genomic regions of interest.

## Background

Multipoint variance component linkage analyses are a commonly used tool of statistical geneticists attempting to locate genes influencing complex phenotypes. More recently, bivariate multipoint linkage methods have been developed to examine linkage for two traits of interest simultaneously [1]. Here, we introduce an extension of this approach that allows for the simultaneous examination of linkage between chromosomal markers and up to three quantitative traits. This trivariate multipoint linkage approach may provide investigators with a greater ability to identify genes having overlapping (pleiotropic) effects on multiple phenotypes.

Several electrophysiological endophenotypes correlated with alcohol dependence have been examined in the Collaborative Study of the Genetics of Alcoholism (COGA). Observed and hypothesized physiological relationships among the endophenotypes suggest pleiotropy may have a role in endophenotype variation. Quantitative trait loci (QTLs) influencing multiple endophenotypes may be detectible using multivariate linkage screens. Thus, the primary objectives of this study were 1) to introduce trivariate linkage screens for quantitative traits, as implemented in SOLAR [2], 2) to apply those methods to three quantitative endophenotypes in the COGA data, and 3) to compare the results of linkage screens conducted with the microsatellite genotypes provided by the Center for Inherited Disease Research (CIDR) to those using genotypes based on the single-nucleotide polymorphisms (SNPs) supplied by Illumina.

## Methods

Details about participant selection and other aspects of COGA can be found in Begleiter et al. [3]. The COGA data provided for the Genetic Analysis Workshop 14 (GAW14) included Visual Oddball event-related potential (ERP) data from 905 genotyped individuals (454 males and 451 females) in 143 extended families [4]. Three quantitative electrophysiological endophenotypes from the target case of the Visual Oddball experiment were selected for analysis: TTTH1, TTTH2, and TTTH3. These represent data extracted from the far frontal left side, frontal midline, and central midline channel electrode placements, respectively. The endophenotypes were measured during the time window 300 to 700 ms following stimulus presentation, and correspond to the theta power band (3 to 7 Hz). We selected TTTH1, TTTH2, and TTTH3 for examination based on preliminary analyses indicating strong linkage signals in univariate linkage screens, and on significant genetic correlations between the trait pairs (see below).

Endophenotype values ± 4 SD from the mean were blanked prior to analysis. After removing outliers, TTTH1 data were available for 901 individuals, TTTH2 data for 902 individuals, and TTTH3 data for 903 individuals. Measured covariates considered included sex, age at electrophysiology data collection (ERPAGE), habitual smoking status (SMOKER), and maximum number of drinks consumed in a 24-hour period (MXDRNK). Whereas the inclusion in models of covariates genetically correlated with traits of interest can reduce analytical power, genetic correlations between MXDRNK and SMOKER and the three endophenotypes examined were minimal (ρ_g _< 0.10), and were not significant (*p *> 0.05).

All statistical genetic analyses used maximum-likelihood methods and were implemented in SOLAR. Because the values of TTTH1 were not normally distributed, we used the multivariate t distribution rather than the multivariate normal distribution in our analyses. Linkage between the autosomal markers and the endophenotype values was assessed using standard multipoint variance components linkage methods extended to allow for simultaneous examination of up to three quantitative traits. Linkage analyses were initially conducted using microsatellite markers supplied by CIDR; analyses were then repeated using genotypes based on the SNP markers provided by Illumina. Genotyping strategies have been detailed elsewhere [4]. Final models were the most parsimonious, and included only the covariates whose contribution to an endophenotype's variance met a threshold value of *p *< 0.10.

Genetic positions were based on map information distributed to GAW14 participants. Multipoint identity-by-descent (MIBD) matrices were generated using LOKI [5]. All LODs were corrected to a standard equivalent to 1 degree of freedom. For bivariate linkage analyses, this follows the logic outlined by Amos et al. [6], and assumes a LOD score distribution of 1/4 point mass at 0, 1/2 χ^2^_1_, and 1/4 χ^2^_3_. Extending this logic, the trivariate LOD was corrected as 1/8 point mass at 0, 3/8 χ^2^_1_, 3/8 χ^2^_3_, and 1/8 χ^2^_6_. For the COGA data provided for GAW14, a LOD threshold equivalent to a genome-wide *p*-value of 0.05 was estimated by the method of Feingold et al. [7] as 2.77 for the microsatellite-based analyses, and 3.36 for the Illumina SNP-based analyses.

The assumptions of linkage equilibrium that underlie multipoint linkage analysis may be violated if genetic markers are densely spaced, thus increasing the likelihood of linkage disequilibrium (LD) between markers. LD between markers is a particular concern with SNP-based maps, which often have higher marker density than occurs in traditional microsatellite-based maps. A specific analysis of the potential impact of LD between the Illumina SNPs on the results of our trivariate linkage analyses was beyond the scope of this paper. However, in another paper [8] we examined the impact of LD between markers on linkage analysis of an electrophysiological endophenotype from the COGA dataset. Despite observing considerable linkage disequilibrium between the Illumina SNPs in the dataset provided for GAW14, we found that correcting for LD resulted in only "modest changes in LOD score magnitude and shifts in the position of the maximum LOD." The direction of the magnitude shift was inconsistent. Based on this, we believe that the effect on our results of uncorrected LD between SNPs is likely to be minor.

## Results

### Heritability and univariate linkage screens

Table 1 presents for each endophenotype the estimated heritability (h^2^, or the proportion of the residual phenotypic variance explained by additive genetic effects), and the covariates meeting the threshold for model inclusion. For TTTH1, a genome-wide linkage scan using the microsatellite genotypes yielded a maximum multipoint LOD of 3.41 at 157 cM on chromosome 7q. Approximately 23 cM qter of this, a locus at 180 cM also has a LOD > 2 (LOD = 2.37). The strongest linkage signal (LOD = 1.80) for TTTH2 was in approximately the same location as that of TTTH1, at 160 cM on chromosome 7q. For TTTH3, the maximum multipoint LOD (2.42) was at 43 cM on chromosome 3p. No other LODs over 2 were estimated for TTTH1, TTTH2, or TTTH3.

Results of linkage analyses conducted using the Illumina SNP genotypes were similar. For TTTH1, we estimated a maximum multipoint LOD of 3.38 at 139 cM on chromosome 7q. A second strong linkage signal (LOD = 3.06) occurred on this same chromosome, at 154 cM. For TTTH2, the strongest linkage signal (LOD = 2.01) was on chromosome 3p at 49 cM. The maximum multipoint LOD score for TTTH3 was 3.67, on chromosome 7q at 174 cM. The next highest LOD in the initial linkage pass for TTTH3 was 2.00, located on chromosome 3p at 53 cM. No other LOD scores over 2 were estimated for any of the three endophenotypes in the univariate linkage analyses using the Illumina SNP-based genotypes.

### Genetic correlations and bivariate linkage screens

Strong genetic (ρ_g _≥ 0.71) and moderate to strong environmental (0.74 ≥ ρ_e _≥ 0.41) correlations occurred between the endophenotypes (Table 2). All correlations were significantly different from zero (*p *< 0.0001). When TTTH1 and TTTH2 were considered simultaneously, a genome-wide multipoint linkage screen using the microsatellite-based genotypes yielded one LOD > 2, a linkage signal with LOD = 3.49 at 158 cM on chromosome 7q. Using the Illumina SNP-based genotypes, we estimated a maximum multipoint LOD of 2.55 for TTTH1 and TTTH2 on chromosome 7q, at 140 cM. A weak linkage signal (LOD = 2.35) also occurred at 87 cM on chromosome 2p.

The location of the strongest bivariate linkage signal for TTTH1 and TTTH3 approximated that of TTTH1 and TTTH2. For both the microsatellite- and the Illumina SNP-based linkage screens, multipoint LODs > 2 occurred only on chromosome 7. Using the microsatellite-based genotypes, we estimated a maximum multipoint LOD of 3.59 at 157 cM on chromosome 7q. The maximum multipoint LOD estimated for TTTH1 and TTTH3 using the Illumina SNP-based genotypes was 2.79, located at 173 cM on chromosome 7q. Additional LODs > 2 occurred on this same chromosome at 139 cM (LOD = 2.74) and 155 cM (LOD = 2.17).

The strongest linkage signal (LOD = 2.15) estimated for TTTH2 and TTTH3 using the microsatellite-based genotypes occurred at 40 cM on chromosome 3p. The next strongest signal (LOD = 1.73) for these endophenotypes occurred at 183 cM on chromosome 7q. By contrast, using the Illumina SNP-based genotypes we estimated a maximum multipoint LOD score of 3.58 on chromosome 7q at 175 cM. No additional LODs over 2 occurred.

### Trivariate linkage screen

**Figure 1:**
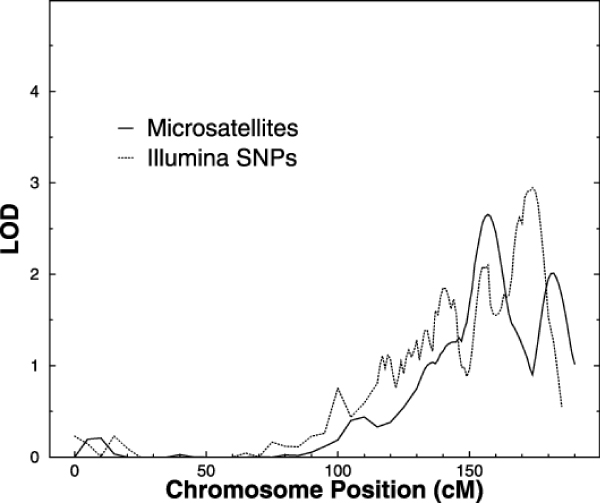
Trivariate multipoint linkage results for chromosome 7.

Using the microsatellite-based genotypes and a genome-wide linkage screen that considered TTTH1, TTTH2, and TTTH3 simultaneously, we estimated a maximum multipoint LOD of 2.66 at 157 cM on chromosome 7q. Results of genome-wide linkage analyses utilizing the Illumina SNP-based genotypes were similar, yielding a maximum multipoint LOD of 2.95 for these endophenotypes at 174 cM on chromosome 7q. Additionally, a LOD of 2.39 occurred on chromosome 2p at 79 cM. No other LODs over 2 were estimated in either the microsatellite- or the Illumina SNP-based linkage screens. The results of the microsatellite- and SNP-based linkage scans of chromosome 7 are compared in Figure 1.

## Conclusion

We have introduced a trivariate multipoint linkage screen, a novel approach investigators can use in SOLAR to examine regions of interest for genes having potential pleiotropic influence on three quantitative traits. Here, we used this approach to examine three electrophysiological traits correlated with susceptibility to alcohol dependence in the COGA families, TTTH1, TTTH2, and TTTH3. Whereas the strongest linkage signals estimated in the trivariate multipoint linkage analyses did not achieve statistical significance (genome-wide *p *> 0.05), we were able to detect regions of interest corresponding to those identified in the univariate and bivariate linkage screens. Based on a comparison of the number LOD scores achieving statistical significance, our results also suggest that the microsatellite- and Illumina SNP-based genotypes have similar utility for detecting genomic regions of interest.

Our results suggest that the trivariate approach has utility in the further characterization of chromosomal regions potentially containing genes influencing the phenotypes being examined. However, additional analyses using other datasets, including simulation studies, are required to evaluate the power of this approach relative to other methods. Similar to bivariate linkage screens [1], trivariate linkage screens may allow for more accurate parameter estimates and better localization than univariate screens. Whereas the use of trivariate rather than multiple univariate screens may reduce the problems associated with multiple testing, the computing time required to conduct these screens is considerable, and preliminary analyses using univariate or bivariate linkage screens may be helpful for prioritizing genomic regions to be examined through a trivariate analysis.

## Abbreviations

CIDR: Center for Inherited Disease Research

COGA: Collaborative Study of the Genetics of Alcoholism

ERP: Event-related potential

ERPAGE: Age at Electrophysiology data collection

GAW14: Genetic Analysis Workshop 14

LD: Linkage disequilibrium

MIBD: Multipoint identity by descent

MXDRNK: Maximum number of drinks consumed in a 24-hour period

QTL: Quanitative trait loci

SMOKER: Habitual smoking status

SNP: Single-nucleotide polymorphism

## Authors' contributions

DMW performed the data analysis and drafted the manuscript. TDD generated the MIBD matrices used in the analyses. CPP provided the computer programs used to implement the analyses. MCM contributed to data analysis. JB developed the multivariate modeling methodology. LA was responsible for study design and implementation, and helped to draft the manuscript. All authors read and approved the final manuscript.

## Figures and Tables

**Table 1:** Estimated heritabilities (h^2^)

Endophenotype	h^2^	SE	Covariates contributing to models
TTTH1	0.40**	0.07	ERPAGE**, ERPAGE^2^†, MXDRNK†
TTTH2	0.59**	0.07	ERPAGE**, ERPAGE^2^†, SEX‡, MXDRNK‡, SMOKER*
TTTH3	0.59**	0.07	ERPAGE**, ERPAGE^2^*, SEX§, MXDRNK‡, SMOKER†

*0.05 ≤ *p *< 0.10; †*p *< 0.05; ‡*p *< 0.01; §*p *< 0.001; **p < 0.0001

**Table 2:** Genetic (ρ_g_) and environmental (ρ_e_) correlations between endophenotypes

Endophenotype	ρ_g_	ρ_e_
TTTH1 and TTTH2	0.88*	0.66*
TTTH1 and TTTH3	0.71*	0.41*
TTTH2 and TTTH3	0.93*	0.74*

*Difference from 0, *p *< 0.0001
